# KCa1.1 and Kv1.3 channels regulate the interactions between fibroblast-like synoviocytes and T lymphocytes during rheumatoid arthritis

**DOI:** 10.1186/s13075-018-1783-9

**Published:** 2019-01-07

**Authors:** Mark R. Tanner, Michael W. Pennington, Satendra S. Chauhan, Teresina Laragione, Pércio S. Gulko, Christine Beeton

**Affiliations:** 1 Department of Molecular Physiology and Biophysics, Baylor College of Medicine, One Baylor Plaza, Houston, TX 77030 USA; 2 Interdepartmental Graduate Program in Translational Biology and Molecular Medicine, Baylor College of Medicine, Houston, TX USA; 3 Peptides International, Inc., Louisville, KY USA; 4 Present address: Ambiopharm, Inc., North Augusta, SC USA; 5 Division of Rheumatology, Department of Medicine, Icahn School of Medicine at Mount Sinai, New York, NY USA; 6 Biology of Inflammation Center, Center for Drug Discovery, Cardiovascular Research Institute, and Dan L Duncan Comprehensive Cancer Center, Baylor College of Medicine, Houston, TX USA

**Keywords:** Synovial fibroblast, Immunomodulation, Cell interactions, Dual therapy, Autoimmunity

## Abstract

**Background:**

Fibroblast-like synoviocytes (FLS) and CCR7^−^ effector memory T (T_EM_) cells are two of the major cell types implicated in the progression of rheumatoid arthritis (RA). In particular, FLS become highly invasive, whereas T_EM_ cells proliferate and secrete proinflammatory cytokines, during RA. FLS and T cells may also interact and influence each other’s phenotypes. Inhibition of the pathogenic phenotypes of both FLS and T_EM_ cells can be accomplished by selectively blocking the predominant potassium channels that they upregulate during RA: KCa1.1 (BK, Slo1, MaxiK, *KCNMA1*) upregulated by FLS and Kv1.3 (*KCNA3*) upregulated by activated T_EM_ cells. In this study, we investigated the roles of KCa1.1 and Kv1.3 in regulating the interactions between FLS and T_EM_ cells and determined if combination therapies of KCa1.1- and Kv1.3-selective blockers are more efficacious than monotherapies in ameliorating disease in rat models of RA.

**Methods:**

We used in vitro functional assays to assess the effects of selective KCa1.1 and Kv1.3 channel inhibitors on the interactions of FLS isolated from rats with collagen-induced arthritis (CIA) with syngeneic T_EM_ cells. We also used flow cytometric analyses to determine the effects of KCa1.1 blockers on the expression of proteins used for antigen presentation on CIA-FLS. Finally, we used the CIA and pristane-induced arthritis models to determine the efficacy of combinatorial therapies of KCa1.1 and Kv1.3 blockers in reducing disease severity compared with monotherapies.

**Results:**

We show that the interactions of FLS from rats with CIA and of rat T_EM_ cells are regulated by KCa1.1 and Kv1.3. Inhibiting KCa1.1 on FLS reduces the ability of FLS to stimulate T_EM_ cell proliferation and migration, and inhibiting Kv1.3 on T_EM_ cells reduces T_EM_ cells’ ability to enhance FLS expression of KCa1.1 and major histocompatibility complex class II protein, as well as stimulates their invasion. Furthermore, we show that combination therapies of selective KCa1.1 and Kv1.3 blockers are more efficacious than monotherapies at reducing signs of disease in two rat models of RA.

**Conclusions:**

Our results demonstrate the importance of KCa1.1 and Kv1.3 in regulating FLS and T_EM_ cells during RA, as well as the value of combined therapies targeting both of these cell types to treat RA.

**Electronic supplementary material:**

The online version of this article (10.1186/s13075-018-1783-9) contains supplementary material, which is available to authorized users.

## Background

Rheumatoid arthritis (RA) is a chronic autoimmune disease featuring inflammation centralized within the synovial joints [1, 2]. Despite major advances in treatment strategies for RA, remission remains uncommon and is achieved in only a subset of patients [3]. Current treatments also render patients immunosuppressed and at increased risk for infections [4]. Therefore, there is an unmet need for novel and innovative strategies to treat this disease without further immunosuppressing patients. T cells have a role in disease pathogenesis; in particular, CD4^+^CD45RA^−^CCR7^−^ effector memory T lymphocytes (T_EM_ cells) are a primary effector T cell population responsible for the inflammatory aspect of this disease, with characteristic phenotypes of being highly proliferative and the secretion of proinflammatory cytokines within the synovium, leading to joint inflammation [5–7]. Following activation, T_EM_ cells upregulate the potassium channel Kv1.3 at their plasma membrane, as opposed to naïve and central memory T cells, which primarily express the KCa3.1 potassium channel [6–8]. Selective blockade of Kv1.3 reduces T_EM_ cell proliferation and cytokine secretion while leaving naïve and central memory T cells able to become activated. Kv1.3-selective blockers are effective at reducing disease severity in multiple animal models of autoimmunity, including in the pristane-induced arthritis (PIA) model of RA, without affecting the clearance of acute infections [6, 7, 9, 10]. As such, Kv1.3 blockers have emerged as promising therapeutics for the treatment of RA [6, 7, 9, 11]. Indeed, ShK-186 (Dalazatide; Kv1.3 Therapeutics, Inc., Seattle, WA, USA), an analog of a venom peptide from the sea anemone *Stichodactyla helianthus*, is a highly potent and selective Kv1.3 blocker that has shown promise in early clinical trials for the treatment of T_EM_ cell-mediated autoimmune disease [12, 13]. As promising Kv1.3 blockade is as an RA therapy, in experimental models of autoimmunity, ShK-186-treated animals still exhibit signs of joint damage, albeit at significantly lower levels than in control vehicle-treated animals [6, 9]. It is likely that other cell types involved in the pathogenesis of RA and its animal models that do not express Kv1.3 are still active following Kv1.3 block and continue to cause disease progression.

Fibroblast-like synoviocytes (FLS) are resident synovial joint cells that develop a highly invasive phenotype during RA, contributing to joint damage, and secrete a variety of proinflammatory cytokines and chemokines [14, 15]. We have previously demonstrated that FLS from patients with RA and from animal models of RA upregulate the potassium channel KCa1.1 at their plasma membrane [16–20]. KCa1.1 blockade with selective inhibitors, such as the fungal alkaloid paxilline and the *Buthus tamulus* scorpion venom toxin iberiotoxin (IbTX), reduces FLS invasion and cytokine and chemokine secretion ex vivo [17, 19, 21]. Furthermore, KCa1.1 blockers reduce disease severity in animal models of RA [17, 21]. However, similar to what is observed in animal models of autoimmunity treated with Kv1.3 blockers, rats with a model of RA treated with a KCa1.1 blocker still exhibit signs of disease, but at lower levels than vehicle-treated animals [17, 21].

FLS and T cells interact within ex vivo settings in which, when stimulated with interferon (IFN)-γ, FLS express major histocompatibility complex (MHC) class II molecules along with the costimulatory molecule B7-H3 (CD276), intercellular adhesion molecule (ICAM)-1 (CD54), and CD40, allowing FLS to serve as antigen-presenting cells to CD4^+^ T cells [22–27]. T_EM_ cells also secrete a variety of cytokines, including tumor necrosis factor (TNF)-α, interleukin (IL)-17, and IFN-γ, that are known to induce or enhance the highly invasive, pathogenic phenotype of FLS [28–30]. Therefore, it is likely that FLS and T_EM_ cells interact during RA to increase each other’s pathogenic features. It may be possible to reduce these interactions through modulating the predominant potassium channels each cell expresses. Importantly, FLS do not express Kv1.3, and the Kv1.3 blocker ShK-186 does not inhibit the RA-FLS pathogenic phenotype, because ShK-186 does not block KCa1.1 channels [19, 31, 32]. Likewise, no T cell populations are known to express KCa1.1, and the KCa1.1 blockers paxilline and IbTX do not block Kv1.3, the potassium channel predominantly expressed by T_EM_ cells [7, 33, 34].

In this study, we show that KCa1.1 is a regulator of MHC class II molecule expression in FLS from the collagen-induced arthritis (CIA) model of RA. KCa1.1 block reduces the CIA-FLS ability to stimulate the proliferation and migration of T_EM_ cells. We further show that blocking Kv1.3 reduces T_EM_ cells’ ability to induce the invasion of CIA-FLS and induce an increase in expression of KCa1.1 and MHC class II molecules on CIA-FLS. Finally, we show that a combined therapy of potassium channel blockers targeting both KCa1.1 and Kv1.3 is more effective than monotherapies at reducing disease severity in two rat models of RA. Our studies highlight the importance of KCa1.1 on FLS and Kv1.3 on T_EM_ cells as moderators of disease severity in RA, and they further validate the use of selective, potent potassium channel blockers as novel therapies for RA.

## Methods

### Animals

All experiments involving rats were approved by the Institutional Animal Care and Use Committee at Baylor College of Medicine. Female Lewis rats (8–11 weeks old; Charles River Laboratories, Wilmington, MA, USA) and female Dark Agouti rats (8–11 weeks old; Envigo, Indianapolis, IN, USA) were housed in autoclaved setups in an Association for Assessment and Accreditation of Laboratory Animal Care International-accredited facility in which they were provided food and water ad libitum.

### Isolation and culture of FLS

FLS from patients with RA, as defined by criteria of the American College of Rheumatology [35], were isolated as described previously [36]. FLS from rats with CIA, induced with disease as described below, were isolated 14 days after the rats developed signs of disease, as described previously [37] by isolating the synovial paw joints, incubating them with Gibco type IV collagenase (Life Technologies, Carlsbad, CA, USA) for 1 h at 37 °C, and culturing adherent cells in DMEM supplemented with 2 mg/ml L-glutamine, 0.1 μg/ml streptomycin, 10 U/ml penicillin, and 10% FBS. CIA-FLS and RA-FLS were considered pure after the third passage of the adherent cells and were used between passages 3 and 10.

### KCa1.1 and Kv1.3 channel blockers

The KCa1.1 blocker paxilline was purchased from Fermentek (Jerusalem, Israel), and the Kv1.3 blocker ShK-186/Dalazatide, synthesized under good manufacturing practice conditions by CSBio (Menlo Park, CA, USA), was a kind gift from Kineta, Inc. (Seattle, WA, USA). The KCa1.1 blocker IbTX was synthesized as described previously [21]. Each batch of blockers was tested for channel block by patch-clamping on HEK 293 cells stably expressing KCa1.1 and on L929 cells stably expressing Kv1.3 [38] using a Port-a-Patch automated patch-clamp system (Nanion, Munich, Germany) as described elsewhere [11, 21]. For all in vitro and in vivo studies, potassium channel blockers were used at concentrations known to significantly inhibit the pathogenic phenotypes of FLS and T_EM_ cells and were chosen on the basis of pharmacokinetic and dose-dependence studies [6, 17, 19].

### Measuring MHC class II molecule, B7-H3, ICAM-1, and CD40 expression levels in CIA-FLS

CIA-FLS were treated with 100 ng/ml recombinant IFN-γ (MilliporeSigma, Burlington, MA, USA) for 72 h in the presence or absence of 20 μM paxilline. To measure levels of MHC class II molecules, cells were scraped from culture dishes and left either intact or permeabilized with 0.5% saponin, followed by staining with an anti-MHC class II molecule antibody (clone HIS19; LSBio, Seattle, WA, USA), recognizing the RT1L haplotype expressed by Lewis rats [39], followed by a secondary antibody labeled with the Alexa Fluor 488 fluorophore (Life Technologies). For measurement of B7-H3, ICAM-1, and CD40 expression levels, cells were scraped from their culture flasks and stained with antibodies against B7-H3 (clone MIH42; BioLegend, San Diego, CA, USA) or CD40 (clone 5c3; BioLegend), followed by Alexa Fluor 488-conjugated secondary antibodies, or ICAM-1 (clone HA58; BD Biosciences, San Jose, CA, USA) conjugated to allophycocyanin (APC). Fluorescence was measured using a FACSCanto II flow cytometer (BD Biosciences) and analyzed using FlowJo software (FlowJo, Ashland, OR, USA).

### Preparation of T_EM_ cell conditioned medium

Primary Lewis rat ovalbumin-specific CD4^+^ Th1 T_EM_ cells were provided by Alexander Flügel (University Medical Center, Göttingen, Germany) and maintained in culture as described previously [40]. T_EM_ cell conditioned medium was prepared by stimulating 3 × 10^6^ T_EM_ cells with 150 × 10^6^ irradiated (30 Gy) Lewis rat thymus-derived antigen-presenting cells, which were obtained by making single-cell suspensions of freshly harvested thymuses of healthy Lewis rats [41] that were loaded with 10 μg/ml ovalbumin (MilliporeSigma) in the presence or absence of 100 nM ShK-186. Supernatants were collected after 48 h of stimulation and stored at −80 °C for later use.

### Measuring ex vivo antigen presentation

CIA-FLS antigen presentation was measured as described elsewhere [26]. CIA-FLS (10,000 cells/well) were plated in 24-well plates and treated for 48 h with 100 ng/ml recombinant IFN-γ with or without 20 μM paxilline. Medium was then changed to one containing IFN-γ, paxilline, and either 10 μg/ml ovalbumin or myelin basic protein as relevant or irrelevant antigens, respectively, and cells were cultured for an additional 72 h. CIA-FLS were then washed; 10,000 ovalbumin-specific T_EM_ cells were added to each well; and cocultures were grown for 72 h in medium containing 10 μg/ml ovalbumin or myelin basic protein, but without IFN-γ or paxilline. Proliferation of the T_EM_ cells was measured by [^3^H]thymidine incorporation as described elsewhere [10, 11, 42].

### Imaging conjugates between CIA-FLS and T_EM_ cells

CIA-FLS were stimulated for 72 h with 100 ng/ml IFN-γ and loaded with 10 μg/ml ovalbumin for 2–3 h in the presence of IFN-γ. The CIA-FLS were then labeled with CellTrace Violet dye (Thermo Fisher Scientific, Waltham, MA, USA), and ovalbumin-specific T_EM_ cells were labeled with carboxyfluorescein succinimidyl ester (CFSE) (Thermo Fisher Scientific). The CIA-FLS were then removed from their culture flasks and placed in 1.5-ml Eppendorf tubes with the ovalbumin-specific T_EM_ cells at a 4:1 ratio of T_EM_ cells to CIA-FLS. The cocultures were briefly centrifuged, incubated at 37 °C for 30 min in the presence or absence of either 20 μM paxilline or 100 nM ShK-186, and fixed with 4% paraformaldehyde. Cells were then stained with an anti-CD3 antibody conjugated to APC (antibody clone 1F4; BD Biosciences) and analyzed with an Amnis Imaging Flow Cytometer (MilliporeSigma) to image cell conjugates and the formation of an immune synapse or with a FACSCanto II flow cytometer to quantify cell conjugates, with gating completed to exclude single cells.

### Measuring ex vivo T cell migration

CIA-FLS (5000 cells/well) were plated in 24-well plates and stimulated for 24 h with 100 ng/ml IFN-γ with or without 20 μM paxilline. Medium was then changed, and Transwell inserts with 5-μm pores (Corning, Corning, NY, USA) were placed on top of the CIA-FLS-containing wells. Fifty thousand Lewis rat T_EM_ cells were placed in the Transwell, and T cells were allowed to migrate toward the FLS. After 6 h, the number of T cells that migrated through the Transwell and into the well containing the CIA-FLS was counted with a hemocytometer, as described previously [43].

### Measuring ex vivo FLS invasion toward T cell supernatants

CIA-FLS invasion assays were completed as previously described [17, 19, 36, 37]. Briefly, CIA-FLS were placed in the top well of a Matrigel-coated Transwell insert (Corning) in serum-free culture medium. Culture medium containing 20% T cell conditioned medium or 10% FBS was placed in the well beneath the Transwell, and CIA-FLS were allowed to migrate for 24 h. The Matrigel and noninvading cells were then removed, and cells that invaded through the Transwell were counted.

### Measuring the effect of T cells or recombinant cytokines on FLS MHC class II and KCa1.1 expression

CIA-FLS (10,000 cells/well) were plated in 96-well plates and incubated for 72 h in T_EM_ cell-conditioned medium supplemented with 10% FBS. For RA-FLS, cells were stimulated for 24 h in the presence or absence of 100 ng/ml recombinant TNF-α (Bachem, Bubendorf, Switzerland), IL-1β (R&D Systems, Minneapolis, MN, USA), receptor activator of nuclear factor κΒ ligand (RANKL) (MilliporeSigma), or IFN-γ (MilliporeSigma). The FLS were then analyzed by flow cytometry for the plasma membrane expression of MHC class II molecules, as described above. For detection of KCa1.1 levels, cells were permeabilized with 0.5% saponin and stained with an anti-KCa1.1α subunit antibody (clone L6/60; NeuroMab/UC Davis, Davis, CA, USA) followed by an Alexa Fluor 488-conjugated secondary antibody, with analysis by flow cytometry.

### Inducing, monitoring, and treating rat models of RA

CIA was induced in Lewis rats by a subcutaneous injection at the base of the tail with 200 μl of a 1:1 emulsion of 2 mg/ml porcine type II collagen (Chondrex, Redmond, WA, USA) with incomplete Freund’s adjuvant. Seven days after the first injection, rats were given a booster of 100 μl of the collagen and adjuvant emulsion [21, 44]. PIA was induced in Dark Agouti rats by a subcutaneous injection of 150 μl of pristane (MP Biomedicals, Santa Ana, CA, USA) at the base of the tail [6, 17, 21]. Disease onset was defined as development of at least one swollen or red paw joint. Clinical scores were determined daily as described previously [6, 9, 17] by assigning 1 point for each swollen or red toe joint and 5 points for each swollen wrist or ankle, giving each rat a maximum possible score of 60. Upon disease onset, rats were treated every other day with vehicle (P6N buffer; 10 mM sodium phosphate, 0.8% NaCl, 0.05% polysorbate 20, pH 6.0) [9], 20 mg/kg paxilline through intraperitoneal injection, 0.5 mg/kg IbTX subcutaneously, 0.1 mg/kg ShK-186 subcutaneously, or a combination of 0.1 mg/kg ShK-186 and either 20 mg/kg paxilline or 0.5 mg/kg IbTX, doses known to reduce disease severity in rat models of RA and chosen on the basis of pharmacokinetic studies and dose-dependence studies [6, 17, 21]. Randomization of rats to treatment groups was completed in which every fourth rat that developed signs of disease on a given day was placed in the same treatment group, thereby disregarding differences in basal disease severity on the day each rat developed signs of disease and accounting for differences in the time between immunization and when a rat developed signs of disease.

### Measuring KCa1.1 expression by FLS from rats with CIA

FLS were isolated from rats with CIA in each treatment group 14 days after disease onset. They were permeabilized with 0.5% saponin and stained with an anti-KCa1.1α antibody (NeuroMab), followed by an Alexa Fluor 488-conjugated secondary antibody, and cells were analyzed by flow cytometry as described above. Linear regression analysis between KCa1.1α staining intensity of FLS from individual rats and each rat’s clinical score on the day of cell collection was completed with Prism software (GraphPad Software, La Jolla, CA, USA).

### T cell phenotyping of lymph node cells of rats with CIA

The draining inguinal lymph nodes were collected from rats with CIA in each treatment group 14 days after disease onset. Single-cell suspensions were made of each lymph node and stained for expression of CD3 conjugated to APC (clone 1F4; BD Biosciences), CD4 conjugated to phycoerythrin (PE)-cyanine 7 (clone W3/25; BioLegend), CD8 conjugated to PE (clone OX-8; BD Biosciences), CD25 conjugated to fluorescein isothiocyanate (clone OX-39; BD Biosciences), and CD45RC conjugated to biotin (clone OX-22; Thermo Fisher Scientific) and further stained with streptavidin conjugated to Alexa Fluor 405. Cells were analyzed by flow cytometry as described above.

### X-rays and histology

Rats with either PIA or CIA were killed after either 21 or 14 days of treatment, and their paws were collected for histology and x-ray analysis. X-rays were completed using an In-Vivo Xtreme Imaging System (Bruker BioSpin, Billerica, MA, USA) on the hind paws and were used by an investigator blinded to treatment groups to assess the presence or absence of abnormal bone structures and erosions. Following collection, hind paws were fixed, decalcified, embedded in paraffin, sectioned, and stained with either H&E or Safranin O/Fast Green. Images of synovial paw joints were taken with an Olympus BX41 microscope equipped with an Olympus Q Color 5 camera at 10× magnification (Olympus, Center Valley, PA, USA). Scoring of disease parameters from histology was completed by an investigator blinded to treatment groups, as described elsewhere [45], in which immune infiltrates, pannus extensions, hyperplasia, and cartilage erosions were quantified by giving a score of 0 when absent, a score of 1 when mild, a score of 2 when moderate, and a score of 3 when a severe amount of these parameters was present.

### Serum chemistry and cytokine analyses

At the end of the CIA trial, blood was collected by terminal cardiac puncture from each rat, and serum was stored at −80 °C. Blood chemistry panels were completed on five randomly selected rats per group through the Center for Comparative Medicine at Baylor College of Medicine by investigators blinded to each rat’s treatment. Serum cytokine analyses were completed on five or six randomly selected rats per group by using a rat cytokine array (Eve Technologies, Calgary, AB, Canada) in a blinded manner.

### Statistical analysis

All statistical analyses were completed by using the Mann-Whitney *U* test or Wilcoxon matched-pairs signed-rank test, except for the CIA and PIA clinical scores analyses, which were completed using repeated measures one-way analysis of variance with Bonferroni post hoc test. All data are shown as mean ± SEM, and *p* values less than 0.05 were considered significant. All statistical analyses were completed using Prism software.

## Results

### KCa1.1 regulates the ability of CIA-FLS to activate and attract T_EM_ cells

**Fig. 1:**
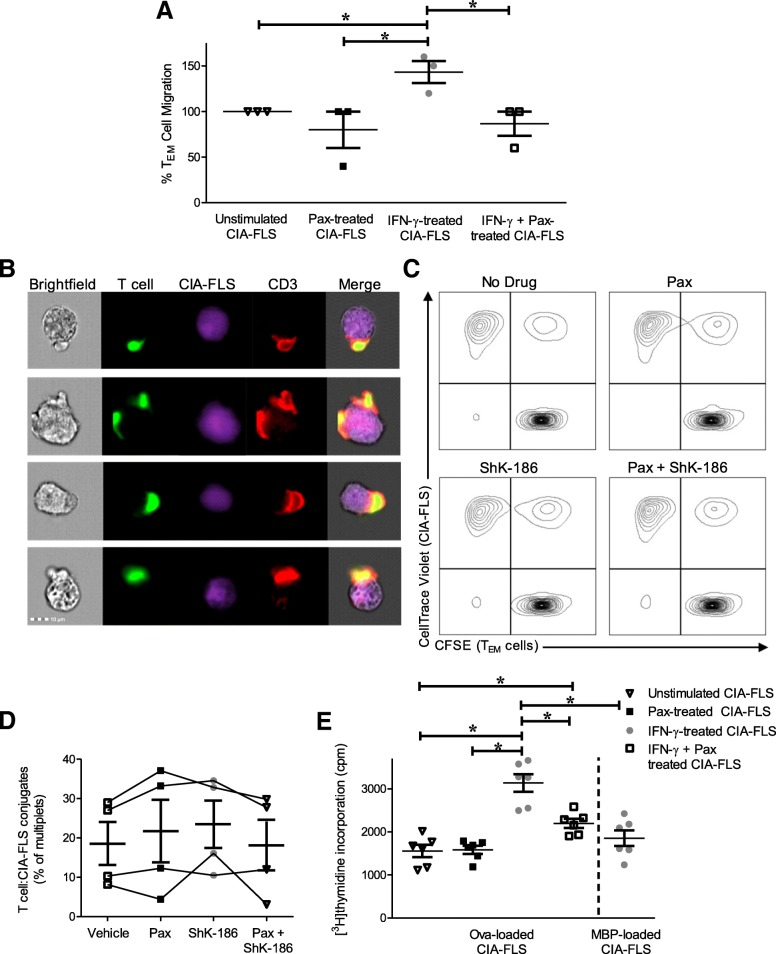
Collagen-induced arthritis fibroblast-like synoviocytes (CIA-FLS) modulate T cell migration and serve as antigen-presenting cells in a KCa1.1-dependent manner. **a** Effector memory T (T_EM_) cell migration toward CIA-FLS that were cultured for 72 h in the presence or absence of interferon (IFN)-γ, paxilline, or both. Data are presented as mean ± SEM (*n* = 3). **b** Flow cytometric images of ovalbumin-loaded, IFN-γ-stimulated CIA-FLS (*violet*) and ovalbumin-specific T_EM_ cell (*green*) cocultures that were stained for CD3 (*red*) after cells were allowed to interact for 30 min. **c** Flow cytometric plots of mixtures of ovalbumin-loaded, IFN-γ-stimulated CIA-FLS (CellTrace Violet) and ovalbumin-specific T_EM_ cells (carboxyfluorescein succinimidyl ester [CFSE]) that were allowed to interact for 30 min in the presence or absence of paxilline (Pax) or ShK-186. Flow cytometric gating was completed to exclude single cells. **d** Quantifications of the proportion of CIA-FLS and T_EM_ cells that form conjugates, as defined by the proportion of CellTrace Violet^+^CFSE^+^ multiplets or nonsingle cells detected by flow cytometry, from experiments in (**c**). Data are presented as mean ± SEM (*n* = 4 separate experiments). **e** Proliferation of cocultures of CIA-FLS with ovalbumin-specific CD4^+^ T_EM_ cells in which CIA-FLS were pretreated with IFN-γ, paxilline (Pax), or both and loaded with either ovalbumin (OVA) or myelin basic protein (MBP). Data are presented as mean ± SEM (*n* = 6). *p < 0.05

Upon cytokine stimulation, FLS secrete chemokines that attract T cells, including CXCL10, IL-6, and fractalkine [46–49]. We sought to determine if KCa1.1 regulates the ability of FLS to stimulate T cell migration. To do this, we treated FLS isolated from Lewis rats with CIA with paxilline and IFN-γ and then used Transwells to monitor syngeneic CD4^+^ T_EM_ cell migration toward the CIA-FLS. T_EM_ cells migrated in equivalent amounts toward unstimulated CIA-FLS and CIA-FLS that had been treated for 72 h with paxilline. However, significantly more T_EM_ cells migrated toward the CIA-FLS that had been pretreated with IFN-γ for 72 h. This effect was eliminated when the CIA-FLS were treated with both IFN-γ and paxilline, indicating that KCa1.1 block influences the ability of IFN-γ-activated CIA-FLS to induce T_EM_ cell migration (Fig. 1a).

Besides attracting T cells toward them, FLS can stimulate the proliferation of CD4^+^ T cells by acting as antigen-presenting cells [26]. To confirm the antigen-presenting capability of FLS, we first verified that CIA-FLS physically bind to and form cell-cell conjugates with CD4^+^ T_EM_ cells. CIA-FLS were stimulated with IFN-γ for 72 h, loaded with ovalbumin, and labeled with the CellTrace Violet fluorescent dye. They were allowed to interact with ovalbumin-specific Lewis rat CD4^+^ T_EM_ cells labeled with the CFSE fluorescent dye. We observed physical interactions between CIA-FLS and T_EM_ cells, with CD3 accumulation observed at the contact site between the CIA-FLS and T_EM_ cells (Fig. 1b), suggesting formation of an immune synapse between the two cell types [50, 51]. To determine if either KCa1.1 or Kv1.3 block prevents the formation of cell conjugates, either paxilline or ShK-186 was added to the mixtures of IFN-γ-treated CIA-FLS and T_EM_ cells while the cells were allowed to interact. Neither paxilline nor ShK-186, alone or in combination, acutely altered the proportion of cell conjugates (Fig. 1c and d).

To confirm that FLS can stimulate the proliferation of CD4^+^ T cells and determine if KCa1.1 block reduces the FLS ability to stimulate T cell proliferation, CIA-FLS were stimulated with IFN-γ in the presence or absence of paxilline, loaded with ovalbumin, and cocultured with syngeneic ovalbumin-specific CD4^+^ T_EM_ cells. The cocultures of ovalbumin-specific T_EM_ cells with CIA-FLS stimulated with IFN-γ and loaded with ovalbumin exhibited significantly greater proliferation than the cocultures containing T cells with CIA-FLS stimulated with IFN-γ in the presence of paxilline and loaded with ovalbumin. Loading IFN-γ-stimulated CIA-FLS with an irrelevant antigen (myelin basic protein) failed to induce the proliferation of the ovalbumin-specific T_EM_ cells (Fig. 1e).

### KCa1.1 regulates surface expression of MHC class II by FLS

**Fig. 2:**
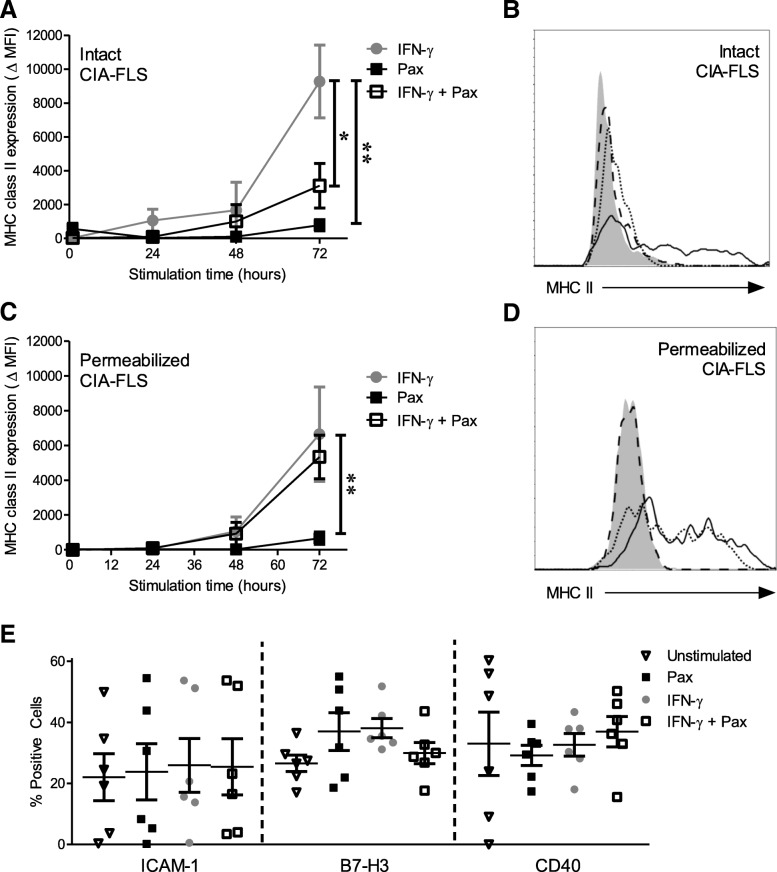
Collagen-induced arthritis fibroblast-like synoviocytes (CIA-FLS) express major histocompatibility complex (MHC) class II molecules that are regulated through KCa1.1, as well as antigen-presenting proteins*.* **a** CIA-FLS plasma membrane expression of MHC class II protein following stimulation for 72 h with IFN-γ (*gray circles*), paxilline (Pax; *black squares*), or interferon (IFN)-γ and paxilline (*open squares*). Data are presented as mean ± SEM (*n* = 3–8 CIA-FLS donors). **b** Flow cytometric histogram of CIA-FLS stained for the plasma membrane expression of MHC class II protein in cells treated with paxilline for 72 h (*dashed line*), stimulated for 72 h with IFN-γ (*solid line*), or stimulated for 72 h with IFN-γ and paxilline (*dotted line*). The shaded histogram represents background staining. **c** MHC class II molecule expression of CIA-FLS that were permeabilized with saponin prior to staining for MHC class II in cells treated for 72 h with IFN-γ (*gray circles*), paxilline (*black squares*), or IFN-γ and paxilline (*open squares*). Data are presented as mean ± SEM (*n* = 3–6 CIA-FLS donors). **d** Flow cytometric histogram of CIA-FLS stained for MHC class II molecules following permeabilization with saponin in cells treated for 72 h with IFN-γ (*solid line*), paxilline (*dashed line*), or IFN-γ and paxilline (*dotted line*). The shaded histogram represents background staining. **e** Expression of intercellular adhesion molecule (ICAM)-1, B7-H3, and CD40 by CIA-FLS following treatment for 72 h with IFN-γ, paxilline, or IFN-γ and paxilline. Data are presented as mean ± SEM (*n* = 6 CIA-FLS donors). *p < 0.05, **p < 0.01

IFN-γ stimulates MHC class II molecule expression by RA-FLS [22, 23, 26]. In order to determine if KCa1.1 regulates this upregulation, CIA-FLS were treated with IFN-γ in the presence or absence of the KCa1.1 blocker paxilline. IFN-γ-treated CIA-FLS had an increase in the total and plasma membrane expression of MHC class II molecules. Examination of intact CIA-FLS indicates that although IFN-γ induces an increase in MHC class II molecules over 72 h, as others have described with RA-FLS [22, 23, 26], cotreatment with paxilline prevents this increase (Fig. 2a and b). However, staining for MHC class II in permeabilized CIA-FLS indicates that paxilline treatment does not reduce the total amount of MHC class II protein expressed, as compared with IFN-γ-treated cells (Fig. 2c and d). Together, these data demonstrate that KCa1.1 block with paxilline reduces the plasma membrane localization of MHC class II molecules in CIA-FLS, but it does not influence the total amount of MHC class II protein synthesized following IFN-γ treatment.

RA-FLS also express the costimulatory molecules B7-H3, ICAM-1, and CD40 [24, 25, 27]. We examined their expression by CIA-FLS following stimulation with IFN-γ in the presence or absence of paxilline. All of these proteins were present in CIA-FLS, and neither IFN-γ nor paxilline had an effect on the proportion of cells expressing them (Fig. 2e). This result was confirmed in RA-FLS (data not shown). We also observed no differences in the expression levels of these proteins between treatment groups, as determined by mean fluorescence intensity, in both RA-FLS and CIA-FLS (data not shown).

### T_EM_ cells and Kv1.3 regulate ex vivo phenotype of CIA-FLS

**Fig. 3:**
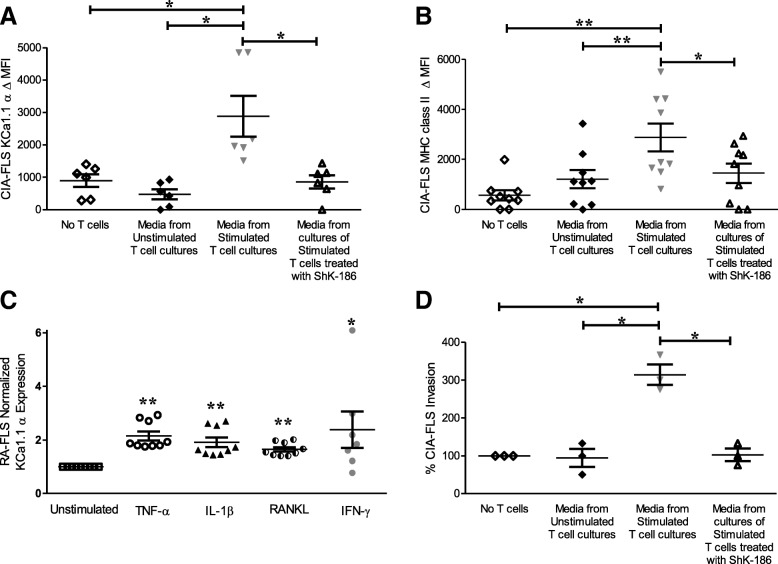
Effector memory T (T_EM_) cells and cytokines regulate the phenotype of fibroblast-like synoviocytes (FLS). KCa1.1α (**a**) and major histocompatibility complex (MHC) class II molecule (**b**) plasma membrane expression levels by collagen-induced arthritis (CIA)-FLS cultured for 72 h in the conditioned medium of T_EM_ cells that were antigen-stimulated in the presence or absence of ShK-186. Data are presented as mean ± SEM (*n* = 6–9 CIA-FLS donors). **c** KCa1.1α protein expression by rheumatoid arthritis (RA)-FLS, as determined by flow cytometry, following 24 h of stimulation with recombinant cytokines. Data are presented as mean ± SEM (*n* = 7–9 RA-FLS donors). **d** CIA-FLS invasion through Matrigel-coated Transwells toward the conditioned medium of T_EM_ cells that were antigen-stimulated in the presence or absence of ShK-186. Data are presented as mean ± SEM (*n* = 3 CIA-FLS donors). *p < 0.05, **p < 0.01

A variety of cytokines, including IFN-γ, IL-17, and TNF-α, are known to influence the activation of FLS [28–30]. It is therefore likely that T_EM_ cells and the cytokines they secrete following activation can directly influence the aggressive phenotype of FLS. ShK-186 treatment prevents T_EM_ cell activation and T_EM_ cells’ ability to secrete a number of cytokines, including IL-2, IL-4, and IFN-γ [6, 52]. We therefore sought to determine if cytokines secreted by T_EM_ cells can influence the activation of FLS and if Kv1.3 blockade on T_EM_ cells influences FLS activation. CIA-FLS cultured in conditioned medium from antigen-stimulated syngeneic CD4^+^ T_EM_ cells expressed significantly higher levels of KCa1.1α (Fig. 3a) and MHC class II molecules (Fig. 3b) at their plasma membrane than CIA-FLS grown in conditioned medium from unstimulated T_EM_ cells. However, CIA-FLS cultured in the conditioned medium from T_EM_ cells that were stimulated in the presence of ShK-186 had significantly reduced MHC class II and KCa1.1α expression compared with those grown in conditioned medium of T_EM_ cells stimulated in the absence of ShK-186 (Fig. 3a and b). These results suggest that the cytokines secreted by activated T_EM_ cells can influence FLS expression of MHC class II and KCa1.1 and that Kv1.3 blockade reduces the T_EM_ cells’ ability to do so. To determine whether cytokines alter KCa1.1 expression in human FLS, we stimulated RA-FLS with either recombinant TNF-α, IL-1β, RANKL, or IFN-γ and assessed them for the expression of KCa1.1α. Compared with unstimulated RA-FLS, those treated with either of these cytokines exhibited increased KCa1.1α expression (Fig. 3c).

Invasiveness is a hallmark of aggressive FLS during RA and its animal models and is enhanced by proinflammatory cytokines such as IFN-γ, IL-17, and TNF-α [28–30]. We therefore examined the influence of the T_EM_ cell conditioned medium on CIA-FLS invasion through Matrigel-coated Transwell inserts. CIA-FLS exposed to conditioned medium from antigen-stimulated T_EM_ cells were significantly more invasive than those exposed to medium from unstimulated T_EM_ cells, indicating that activated T_EM_ cells can enhance CIA-FLS invasion. However, CIA-FLS exposed to conditioned medium of T_EM_ cells stimulated in the presence of ShK-186 were significantly less invasive (Fig. 3d), indicating that Kv1.3 activity regulates the T_EM_ cell ability to induce FLS invasiveness.

### A combined therapy of Kv1.3 and KCa1.1 blockers is more effective than monotherapies in reducing disease severity in CIA

We previously found that monotherapies with either Kv1.3 or KCa1.1 blockers reduce disease severity in rat models of RA, but that each monotherapy does not completely stop disease [6, 7, 11, 17, 21]. This is presumably due to each monotherapy only directly inhibiting either T_EM_ cells or FLS while leaving other pathogenic cell types intact. We therefore sought to determine if a combined therapy of Kv1.3 and KCa1.1 blockers could work in synergy to further ameliorate disease severity in animal models of RA.

**Fig. 4:**
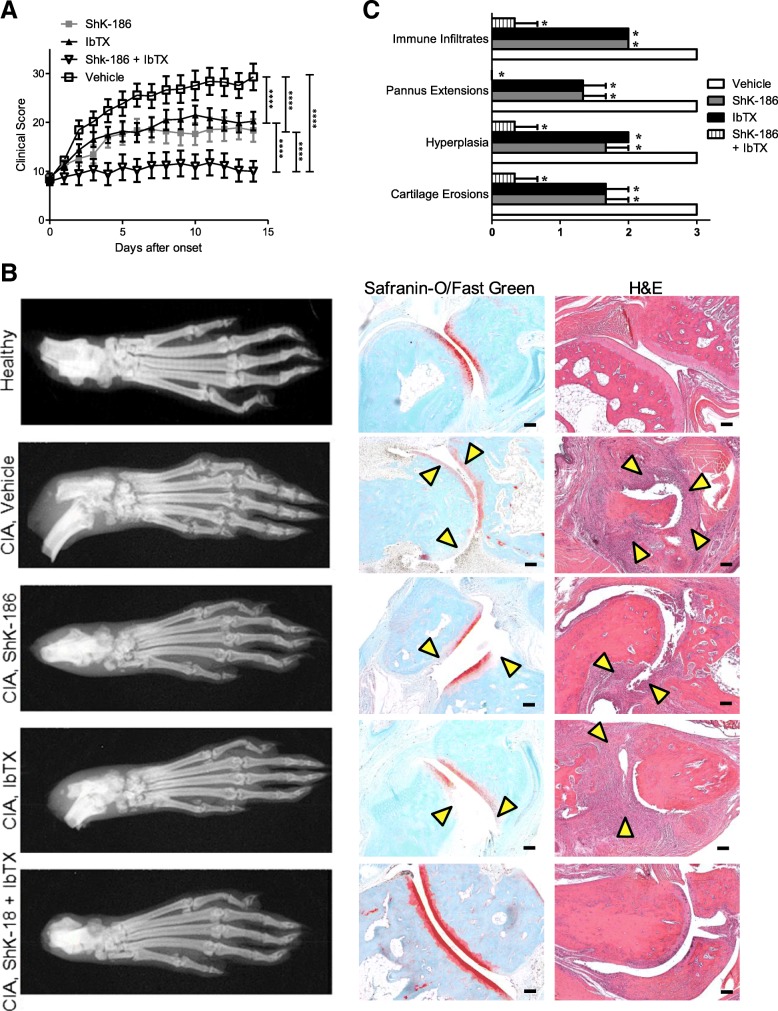
Kv1.3 and KCa1.1 blockers synergize to reduce disease severity in collagen-induced arthritis (CIA). **a** Clinical scores of rats with CIA treated with vehicle (*open squares*), iberiotoxin (IbTX; *black triangles*), ShK-186 (*gray squares*), or both IbTX and ShK-186 (*open triangles*) every other day (EOD) starting at disease onset. Data are presented as mean ± SEM (*n* = 11 or 12 rats per group). **b** *Left*: X-rays of hind paws from a healthy rat and from rats with CIA treated with vehicle, IbTX, ShK-186, or both IbTX and ShK-186 EOD for 14 days after disease onset. *Center* and *right*: Safranin O/Fast Green staining (*center*) and H&E staining (*right*) of tissue sections of hind paw joints of a healthy rat and of rats in each treatment group. *Arrows* indicate areas of cartilage erosions (Safranin O/Fast Green) or hyperplasia (H&E). Scale bar = 100 μm. **c** Scoring of disease parameters from tissue sections of paws from rats of each treatment group (*n* = 3 rats per group). *p < 0.05, ****p < 0.0001

Upon disease onset, rats with CIA were treated with vehicle, the Kv1.3 blocker ShK-186, the KCa1.1 blocker IbTX, or both ShK-186 and IbTX every other day. Vehicle-treated rats developed clinical scores of 29 ± 3 (mean ± SEM), whereas rats treated with either of the monotherapies of ShK-186 and IbTX developed scores of 18 ± 2 and 20 ± 2, respectively, indicating an approximately 30–40% decrease in disease severity in the monotherapy-treated animals. In contrast, rats treated with a combined therapy of ShK-186 and IbTX exhibited significantly less inflammation, with clinical scores of only 10 ± 2, or an approximately 65% reduction in disease severity. Furthermore, whereas vehicle-treated and monotherapy-treated rats exhibited an increase in the clinical scores over time, rats treated with the combined therapy displayed stable, low clinical scores over the course of our studies (Fig. 4a).

X-rays of the hind paws of rats from each treatment group indicated that vehicle-treated rats developed significant bone erosions around the synovial joints, which were reduced by both the monotherapies and the combined therapy (Fig. 4b). Safranin O/Fast Green staining of tissue sections from paw joints in each treatment group demonstrated fewer cartilage erosions in the synovial joints of monotherapy-treated rats and in the combined therapy animals. H&E staining showed that immune infiltrates were reduced in both the monotherapy-treated and combined therapy-treated rats (Fig. 4b and c). We also collected serum from CIA rats in each treatment group 14 days after disease onset and from healthy rats for serum chemistry analyses, which are summarized in Additional file 1: Table S1.

**Fig. 5:**
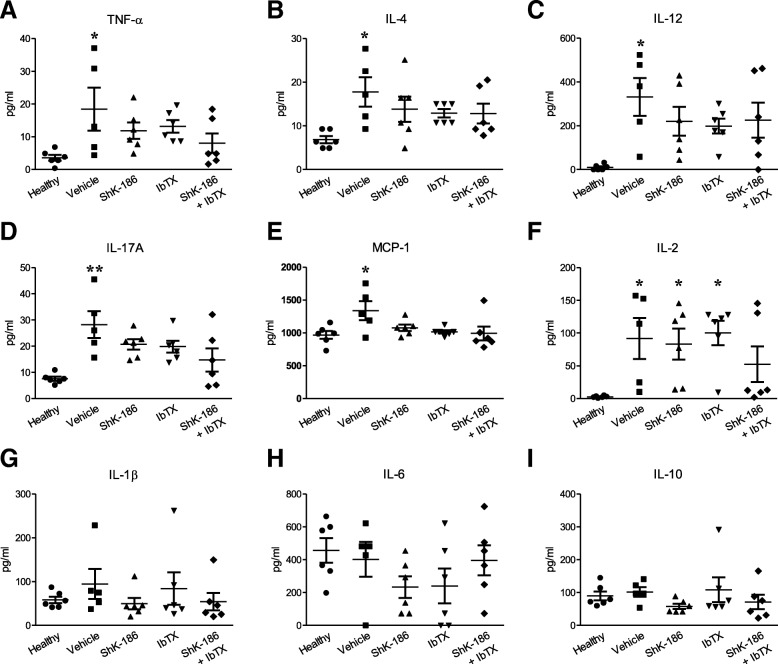
Potassium channel blockers alter serum cytokines in collagen-induced arthritis (CIA). **a**–**i** Serum concentrations of tumor necrosis factor (TNF)-α, interleukin (IL)-4, IL-12, IL-17A, monocyte chemoattractant protein (MCP)-1, IL-2, IL-1β, IL-6, and IL-10 from healthy rats or rats with CIA treated with vehicle, ShK-186, iberiotoxin (IbTX), or both ShK-186 and IbTX every other day for 14 days after disease onset. Data are presented as mean ± SEM (*n* = 5 or 6 rats per group). *p < 0.05

Analyses of cytokine levels in the serum of CIA rats from each treatment group that were collected at the end of the in vivo studies and of healthy rats were also completed. IL-4, IL-12, IL-17A, MCP-1, and TNF-α were increased in vehicle-treated CIA rats compared with in healthy rats, whereas there were no significant differences in the levels of these cytokines in CIA rats treated with ShK-186 or IbTX, alone or in combination, when compared with healthy rats (Fig. 5a–e). IL-2 was increased in CIA rats treated with vehicle, ShK-186, or IbTX, though there was no significant differences in IL-2 levels between healthy rats and rats with CIA treated with both ShK-186 and IbTX (Fig. 5f). IFN-γ was reduced in CIA rats treated with either potassium channel blockers, alone or in combination. IL-5 was increased in CIA rats treated with vehicle or either monotherapy of ShK-186 or IbTX. Vascular endothelial growth factor (VEGF) was decreased in CIA rats treated with both ShK-186 and IbTX, but it was not significantly different in healthy rats compared with CIA rats treated with vehicle or a potassium channel blocker monotherapy. Macrophage inflammatory protein (MIP)-1α was decreased in rats with CIA treated with ShK-186 or both IbTX and ShK-186 compared with healthy rats. Fractalkine, granulocyte-macrophage colony-stimulating factor, GRO/keratinocyte chemoattractant, leptin, LIX, MIP-2, and RANTES (regulated upon activation normal T cell expressed and secreted) were decreased in all rats with CIA compared with in healthy rats, regardless of treatment group. We found no differences in the amount of serum epidermal growth factor, granulocyte colony-stimulating factor, eotaxin, IL-1α, IL-1β, IL-6, IL-10, IL-13, IL-18, or IP-10 between healthy rats and rats with CIA (Fig. 5g–i). These data are summarized in Fig. 5 and Additional file 2: Table S2.

### FLS from potassium channel blocker-treated CIA rats have decreased pathogenic phenotypes

**Fig. 6:**
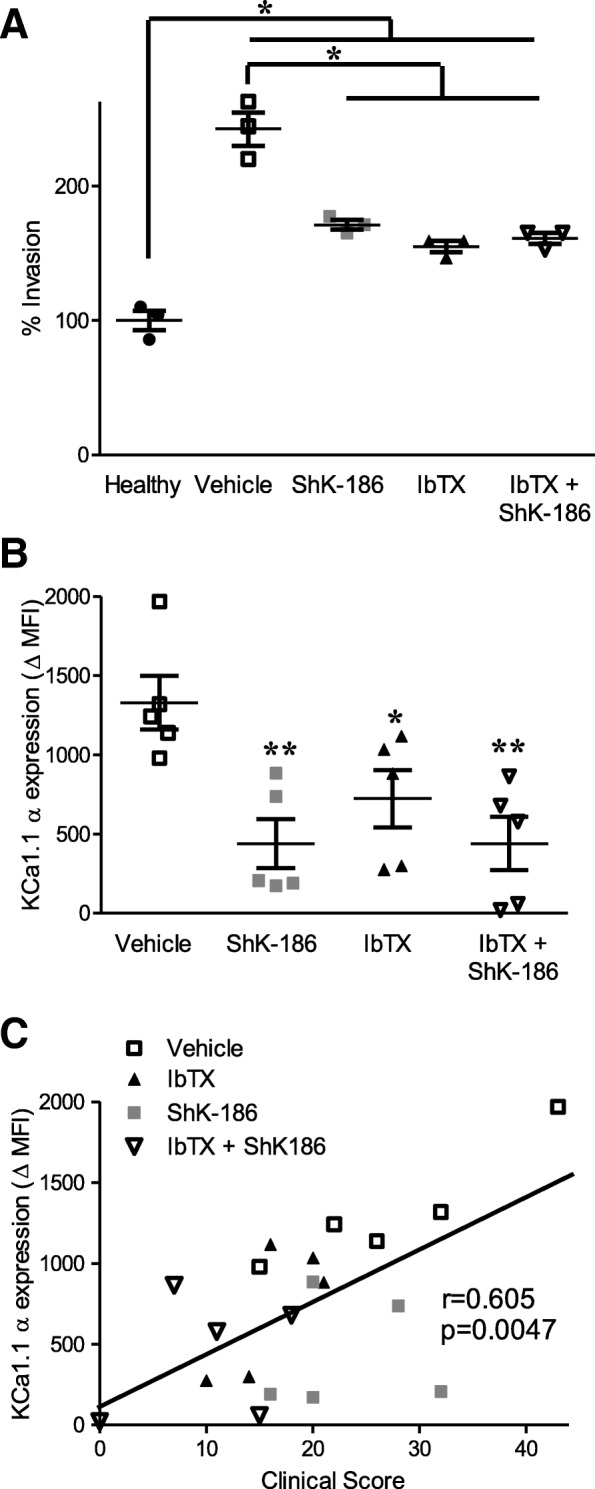
Fibroblast-like synoviocytes (FLS) from collagen-induced arthritis (CIA) rats treated with potassium channel blockers have reduced pathogenic phenotypes ex vivo. **a** Invasion through Matrigel-coated Transwell inserts of FLS isolated from healthy rats or from rats with CIA treated with either vehicle, ShK-186, iberiotoxin (IbTX), or ShK-186 and IbTX every other day (EOD) for 14 days after disease onset. Data are presented as mean ± SEM (*n* = 3 FLS donors per group). **b** KCa1.1α expression by FLS isolated from healthy rats and from rats with CIA treated with vehicle, ShK-186, IbTX, or ShK-186 and IbTX EOD for 14 days after disease onset. Data are presented as mean ± SEM (*n* = 5 FLS donors per group). **c** Data from (**b**) plotted against the clinical scores on the 14th day after disease onset of the rats with CIA from which the FLS were isolated, along with a linear regression plot. *p < 0.05, **p < 0.01

Fourteen days after disease onset, rats with CIA were killed, and FLS were collected from individual rats. FLS isolated from either of the monotherapy or combined therapy treatment groups were significantly less invasive ex vivo than FLS from vehicle-treated rats with CIA, with no differences observed between the three potassium channel blocker treatment groups (Fig. 6a). We previously showed that the level of KCa1.1α expression is directly correlated with FLS invasion and that increasing channel expression increases FLS invasion and decreasing channel expression decreases FLS invasion [17]. We sought to extend this finding in vivo and determined if KCa1.1 expression is correlated with disease severity in CIA. We stained FLS from rats with CIA from each treatment group for KCa1.1α expression and found that FLS from vehicle-treated rats with CIA had significantly higher KCa1.1α expression ex vivo than FLS isolated from rats with CIA treated with ShK-186, IbTX, or both ShK-186 and IbTX (Fig. 6b). Furthermore, through linear regression analyses comparing CIA rat clinical scores with each rat’s FLS KCa1.1α expression, we found that KCa1.1α expression by FLS ex vivo was directly correlated with the disease severity clinical score of the rat from which the FLS were isolated, with an *r* value of 0.605 (Fig. 6c).

### Potassium channel blockers alter T cell populations in CIA rats

**Fig. 7:**
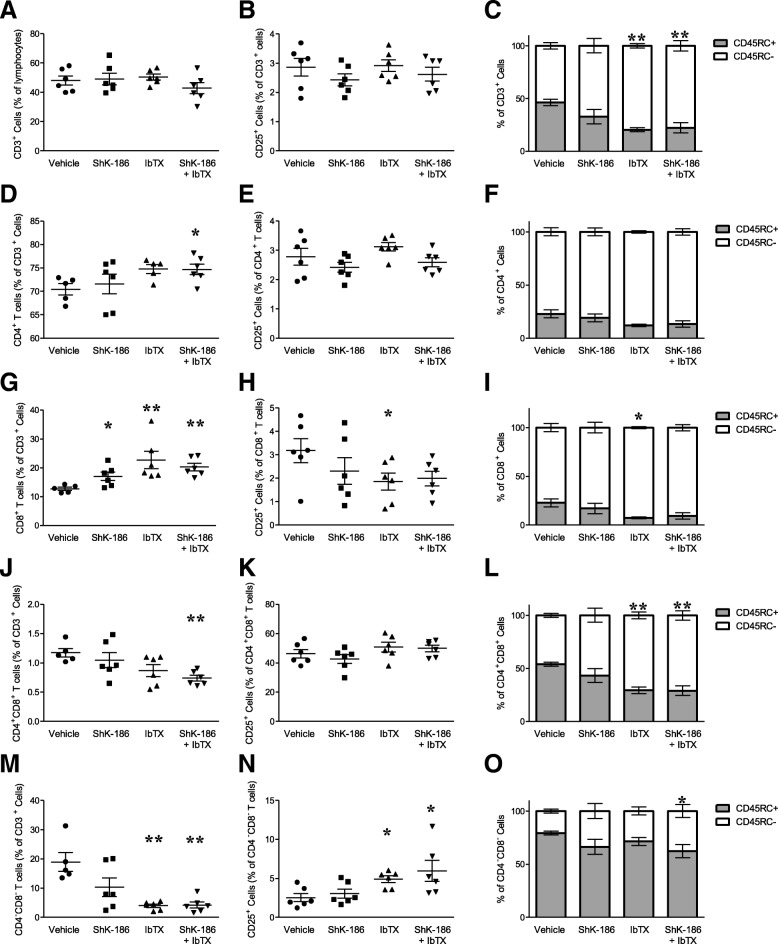
T cell populations are altered in rats with collagen-induced arthritis (CIA) treated with potassium channel blockers. **a**–**o** Expression of CD3, CD4, CD8, CD25, and CD45RC of inguinal lymph node cells of rats with CIA treated with vehicle, ShK-186, iberiotoxin (IbTX), or ShK-186 and IbTX every other day for 14 days after disease onset (*n* = 6 rats per group). *p < 0.05, **p < 0.01

Rats with CIA were killed 14 days after disease onset; draining inguinal lymph nodes were collected; and the populations of T cells were examined using flow cytometry. We found that neither ShK-186 nor IbTX, alone or in combination, altered the total percentage of T cells, as determined by CD3 expression (Fig. 7a) or the percentage of activated T cells (CD3^+^CD25^+^ cells) (Fig. 7b), compared with vehicle-treated CIA rats. However, the proportion of memory T cells (CD3^+^CD45RC^+^) was reduced in the inguinal lymph nodes collected from rats with CIA that were treated with IbTX or both ShK-186 and IbTX compared with those of vehicle-treated CIA rats (Fig. 7c). We also found that the percentage of T cells that were CD4^+^ was increased in the CIA rats treated with both ShK-186 and IbTX (Fig. 7d) compared with those of vehicle-treated rats, though the percentage of CD4^+^ T cells that were CD25^+^ or CD45RC^+^ was not different between groups (Fig. 7e and f). Furthermore, the percentage of T cells that were CD8^+^ was significantly increased in the inguinal lymph nodes of rats with CIA treated with ShK-186, IbTX, or both ShK-186 and IbTX (Fig. 7g). Rats with CIA treated with IbTX had significantly fewer CD8^+^ T cells that were activated (CD25^+^) or memory (CD45RC^+^) cells than those of vehicle-treated CIA rats (Fig. 7h and i). Approximately 1% of the T cells were CD4^+^CD8^+^ and were significantly reduced in the lymph nodes of CIA rats treated with both ShK-186 and IbTX, compared with those treated with vehicle (Fig. 7j). The activation state (CD25 expression) of these cells was unchanged between treatment groups (Fig. 7k), though there were significantly fewer of these cells with a memory phenotype (CD45RC^+^) in rats with CIA treated with IbTX or ShK-186 than in vehicle-treated CIA rats (Fig. 7l). Interestingly, a large proportion of T cells from vehicle-treated CIA rats were CD4^−^CD8^−^. This proportion was reduced in CIA rats treated with IbTX or both ShK-186 and IbTX (Fig. 7m). CD25-expressing CD4^−^CD8^−^ T cells were increased in the CIA rats treated with IbTX or both ShK-186 and IbTX (Fig. 7n), and the percentage of CD45RC-expressing CD4^−^CD8^−^ T cells was also reduced in the CIA rats treated with both ShK-186 and IbTX (Fig. 7o).

### A combined therapy of Kv1.3 and KCa1.1 blockers is more efficacious than monotherapies in treating PIA

**Fig. 8:**
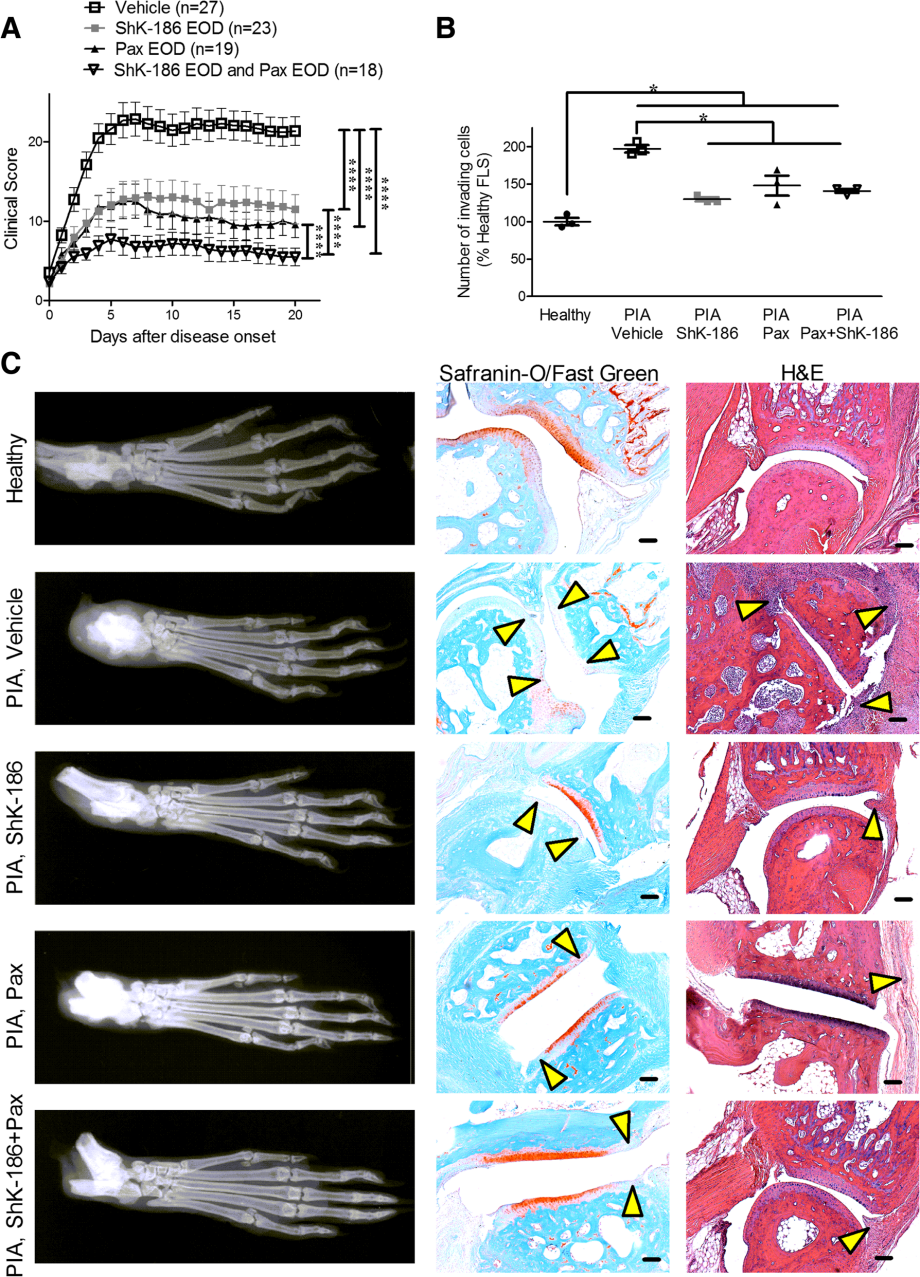
KCa1.1 and Kv1.3 blockers work in tandem to reduce disease severity in pristane-induced arthritis (PIA). **a** Clinical scores of paw inflammation in rats with PIA treated with vehicle (*open squares*), paxilline (Pax; *black triangles*), ShK-186 (*gray squares*), or both paxilline and ShK-186 (*open triangles*) every other day for 21 days after disease onset. Data are presented as mean ± SEM (*n* = 18–27 rats per group). **b** Ex vivo invasiveness of fibroblast-like synoviocytes (FLS) isolated from healthy rats and from rats with PIA treated with vehicle, paxilline, ShK-186, or both paxilline and ShK-186. Data are presented as mean ± SEM, N = 3 FLS donors per group. **c** Left, example X-rays of hind paws of a healthy rat and from rats with PIA treated with vehicle, paxilline, ShK-186, or paxilline and ShK-186 every other day for 21 days after disease onset. *Center* and *right*: Safranin O/Fast Green staining (*center*) and H&E staining (*right*) of tissue sections of hind paw joints from a healthy rat and of rats from each treatment group. *Arrows* indicate areas of cartilage erosions (Safranin O/Fast Green) or hyperplasia (H&E). Scale bar = 100 μm. *p < 0.05, ****p < 0.0001

To further confirm that a combinatorial therapy of Kv1.3 and KCa1.1 blockers is more efficacious at reducing disease severity than monotherapies, we tested the Kv1.3 blocker ShK-186, the KCa1.1 blocker paxilline, or both ShK-186 and paxilline in treating PIA, with treatment starting after disease onset. Similar to the results with CIA, rats with PIA treated with vehicle developed a significant amount of paw inflammation with average scores of 21 ± 2 (Mean ± SEM), whereas those treated with either monotherapies of ShK-186 or paxilline had significantly fewer inflamed joints with scores of 11 ± 2 and 10 ± 2, respectively, an approximately 50% reduction in disease severity. However, rats with PIA treated with both ShK-186 and paxilline had even fewer inflamed joints than monotherapy-treated rats, with clinical scores of 6 ± 1, an approximately 70% reduction in disease severity (Fig. 8a).

Twenty-one days after disease onset, rats with PIA were killed, and FLS were isolated from ankle and toe joints of each rat. Invasion through Matrigel indicated that FLS from the potassium channel blocker-treated PIA rats were significantly less invasive than FLS isolated from vehicle-treated PIA rats (Fig. 8b). Paws were collected, and bone damage was assessed on x-rays. Similar to our results with CIA, rats with PIA treated with vehicle exhibited a significant amount of bone erosion around the synovial joints, which was reduced in the potassium channel blocker-treated rats (Fig. 8c). Safranin O/Fast Green staining and H&E staining of paw joints indicated that cartilage damage and immune infiltrates were reduced in the monotherapy-treated PIA rats, and were even further reduced in the combined therapy-treated PIA rats (Fig. 8c).

## Discussion

**Fig. 9:**
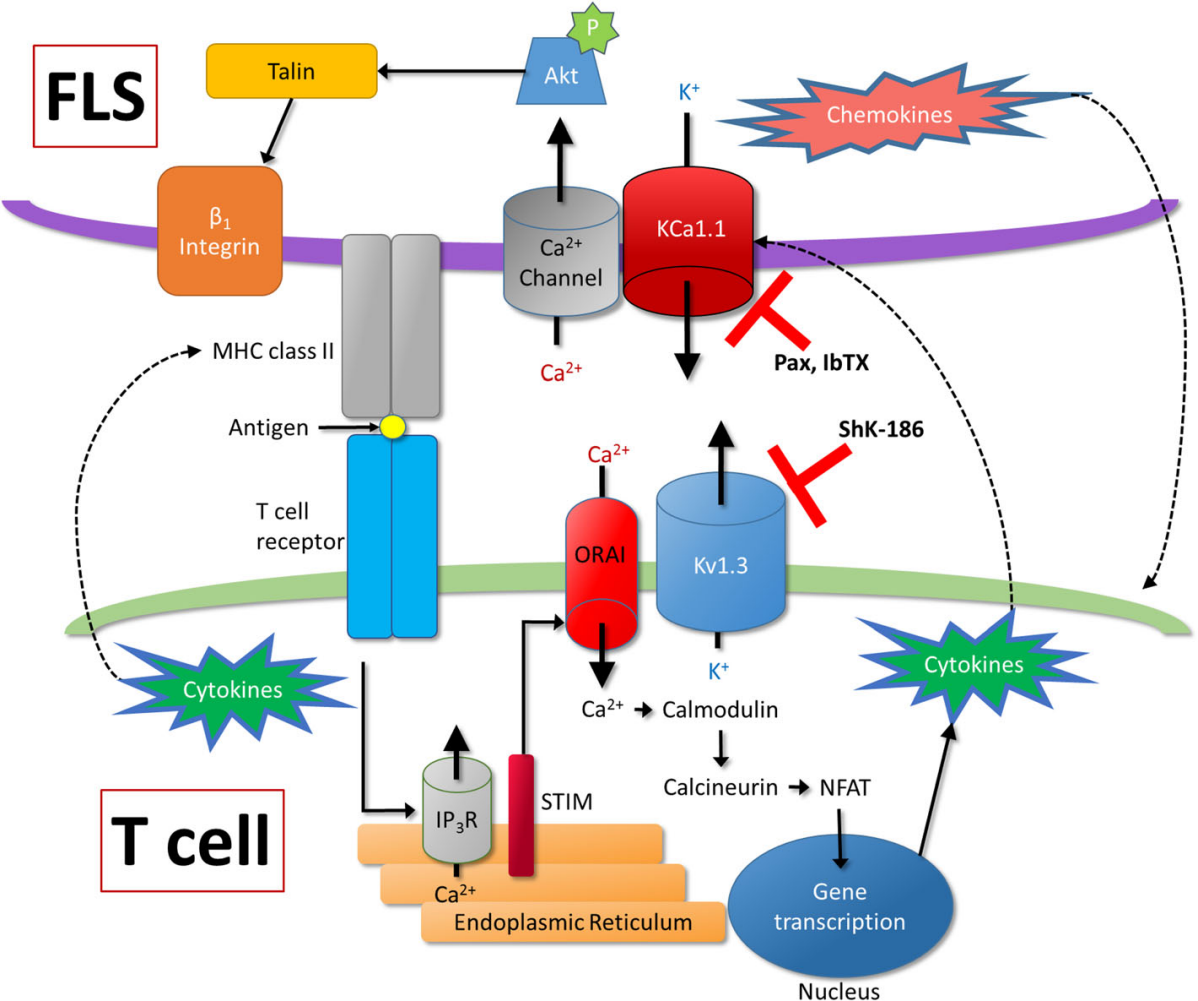
Schematic summarizing the model by which fibroblast-like synoviocytes (FLS) and T cells interact, within the context of previous studies showing the role of KCa1.1 in regulating integrin-mediated invasion in FLS and Kv1.3 in regulating calcium-mediated T cell activation [7, 16]

In this study, we show that FLS from Lewis rats with the CIA model of RA influence the function of Lewis rat T_EM_ cells, and vice versa, and that the major potassium channels expressed by CIA-FLS and by CD4^+^ T_EM_ cells regulate these interactions. These findings are summarized in Fig. 9, where they are shown within the context of previous studies showing the role of KCa1.1 in mediating FLS invasion through modulation of β_1_-integrins and Kv1.3 in regulating calcium-mediated T cell activation [7, 16]. We also show that a combined therapy of potassium channel blockers targeting KCa1.1 expressed by FLS and Kv1.3 expressed by T_EM_ cells work in tandem to reduce disease severity in rat models of RA with more efficacy than monotherapy treatments. Together, these data define the importance of the potassium channels of FLS and T_EM_ cells as regulators of disease in rat models of RA and demonstrate the value of KCa1.1 and Kv1.3 channel blockers as potential therapeutics for RA.

We used FLS isolated from Lewis rats with CIA to examine their in vitro interactions with Lewis rat T_EM_ cells in this study. Others have used human FLS isolated from patients with RA and examined their interactions with T cells isolated from peripheral blood of healthy volunteers with superantigens used to stimulate T cells [23–26], or they have used antigen-specific T cell hybridomas developed from transgenic mice expressing a particular human MHC class II allele [26]. Although using human cells is ideal for the study of disease processes in RA, the mixing of FLS and T cells from different individuals or species could lead to the observation of phenotypes that would not be observed naturally in patients with RA. For this reason, and because of the difficulty in obtaining both FLS and sufficient numbers of T_EM_ cells from the same individuals, we used FLS and T_EM_ cells from inbred Lewis rats. The choice of model species is carefully made because ion channel phenotype, function, and response to pharmacological agents vary between species [32]. We chose rats for this work because the phenotype and function of KCa1.1 in FLS are conserved between humans and rats, as are the phenotype and function of Kv1.3 in T cell subsets in these two species. In contrast, mouse and human T cells diverge in potassium channel expression [32], precluding the use of mice in the present study.

The conditioned medium from activated T_EM_ cells induced an increase in MHC class II and KCa1.1α protein expression in CIA-FLS. In addition, CIA-FLS invaded through Matrigel toward the conditioned medium, indicating that activated T_EM_ cells secrete cytokines which increase the pathogenic features of FLS and chemokines which direct FLS motility. Because the T_EM_ cells used in these experiments are Th1 cells [10], the cytokines responsible for these changes in FLS phenotype were likely IFN-γ or TNF-α, or a combination of both. IFN-γ is the only known cytokine to induce MHC class II expression in FLS [53], whereas both IFN-γ and TNF-α enhance FLS invasion [28–30]. It is likely that one or both of these cytokines induced FLS invasion. These results may also indicate that blocking Kv1.3 can be a novel way to locally interfere with TNF-α production, a key disease mediator, without systemically suppressing a patient’s immune system.

The mechanism by which KCa1.1α expression is regulated in FLS, and how it becomes upregulated during disease, is unknown. However, it is likely through a cytokine-mediated pathway, because our data show that the medium of stimulated T_EM_ cells can induce its upregulation in CIA-FLS and that recombinant proinflammatory cytokines (IFN-γ, TNF-α, and IL-1β) induce KCa1.1α upregulation by RA-FLS. Interestingly, the osteoclastogenic cytokine RANKL, which is secreted by RA-FLS [54], also induces KCa1.1α upregulation in RA-FLS, leading to a possible autocrine or paracrine regulation of KCa1.1. Furthermore, Kv1.3 blockade on T_EM_ cells prevents secretion of some proinflammatory cytokines by RA synovial fluid T_EM_ cells [6]. The conditioned medium of T_EM_ cells stimulated in the presence of the Kv1.3 blocker ShK-186 did not alter FLS invasion, KCa1.1α expression, or MHC class II expression compared with conditioned medium from unstimulated T_EM_ cells. This further verifies that the cytokines secreted by T_EM_ cells alter FLS phenotypes and that Kv1.3 can regulate these phenotypes.

IFN-γ-stimulated CIA-FLS induced the migration of T_EM_ cells toward them, and blocking KCa1.1 on CIA-FLS reduced this effect. This indicates that upon IFN-γ stimulation, CIA-FLS secrete chemokines that influence T cell migration. CIA and RA-FLS are known to produce a variety of T cell chemoattractants upon cytokine stimulation, including CXCL10 [46, 49], fractalkine [48], IL-6, IL-8, CCL2, CCL5, and CXCL12 [47]. KCa1.1 blockade prevents FLS from secreting the chemokines IL-8 and VEGF, but not IL-6 [19]. It is therefore likely that KCa1.1 block also prevents FLS secretion of one or more T cell chemoattractants. In order to determine which particular chemokines are responsible for the FLS-induced T cell migration, it is necessary to first identify the complete milieu of chemokines secreted by IFN-γ-stimulated FLS and determine which of these chemokines are regulated by KCa1.1.

We found that IFN-γ induced an increase in MHC class II protein expression on CIA-FLS, in concordance with previous studies in RA-FLS [23, 26, 53]. Interestingly, cotreatment with the KCa1.1 blocker paxilline reduced the plasma membrane expression of MHC class II molecules, but not the total amount of MHC class II protein present in FLS. These data show that KCa1.1 activity is not necessary for IFN-γ to induce MHC class II protein production, but it is required for its localization to the plasma membrane. The mechanism underlying this observation has yet to be determined, but it may involve KCa1.1 serving as a regulator of protein and vesicle trafficking, cytoskeletal rearrangements, or membrane fluidity and turnover within FLS, which could all result in this channel regulating protein expression at the cell’s surface.

Though stimulating FLS with IFN-γ has been a widely studied means to induce the antigen-presenting phenotype of FLS [22–24, 26, 53], the relevance of FLS antigen presentation and IFN-γ to disease severity and the pathophysiology of RA is subject to debate. Indeed, TNF-α, and not IFN-γ, is generally seen as the prototypical driver of inflammation in RA, and these two cytokines exhibit a mutual antagonism on their effects on FLS phenotypes, including MHC class II protein expression [53]. IFN-γ also reduces IL-1β-induced matrix metalloprotease secretion in FLS, limiting their ability to degrade cartilage [55]. IFN-γ receptor-knockout mice, or mice treated with neutralizing antibodies against IFN-γ, have accelerated onset of CIA, higher disease severity during CIA, and increased Th17 cells [56, 57]. Furthermore, recombinant IFN-γ progressed through several clinical trials as a potential RA therapy [58–60]. However, IFN-γ and its receptor expression are upregulated in the synovium of patients with RA compared with patients with osteoarthritis [61], and IFN-γ promotes FLS motility and invasion [29, 30]. Therefore, it remains to be determined how relevant these observations are within the actual disease state. Furthermore, cytokine receptors can have converging signaling pathways and other proinflammatory cytokines that activate the same signaling cascades as those downstream of the IFN-γ receptor and may induce the same phenotypes observed in FLS following IFN-γ stimulation [62].

Determining if FLS can present antigen on MHC class II and stimulate CD4^+^ T cells in vivo and thereby serve as nontraditional professional antigen-presenting cells may reveal important information regarding inflammation in RA, because it would reveal FLS as an in situ activator of T cells at a site of inflammation. This could have major implications regarding the importance of FLS and IFN-γ in initiating inflammation in RA, because it is possible that localized increases in IFN-γ levels, perhaps from inflammation due to an infection or physical insult to the joint, could activate FLS antigen presentation and therefore initiate T cell-driven inflammation.

We found that several cytokines were elevated in the serum of vehicle-treated rats with CIA compared with healthy rats and that ShK-186 and IbTX normalized their levels. These included IL-4, IL-12, IL-17A, MCP-1, and TNF-α. Given that blocking Kv1.3 on T_EM_ cells reduces their secretion of cytokines, it was expected that ShK-186 treatments would reduce circulating cytokine levels. The mechanisms by which IbTX reduced these cytokines is less clear, though it is possible that inhibiting FLS indirectly causes a reduction in T cell cytokine secretion. Several cytokines were also found in decreased concentrations in the serum of rats with CIA, including fractalkine, leptin, and RANTES, among others. Fractalkine and RANTES are increased in the serum of patients with RA, and their inhibition decreases disease severity in mouse CIA and rat adjuvant-induced arthritis, respectively [63–66]. The mechanisms by which these cytokines decrease in rat CIA remains unknown. Leptin is also associated with RA and is increased in the serum of patients with RA. However, its role in rodent models of RA is less clear and may be decreased in the serum during mouse models of RA [67]. Our data suggest that a similar trend exists in rat models of RA, and overall our data indicate potential divergences in the pathogenesis between RA and its animal models.

We found that rats with either PIA or CIA treated with a Kv1.3 blocker or a KCa1.1 blocker had reduced disease severity, in agreement with previous studies [6, 17]. However, tandem therapies of Kv1.3 and KCa1.1 blockers were even more beneficial than the monotherapies, indicating the value of directly targeting both T_EM_ cells and FLS as a novel and potent therapeutic approach to treating RA. Furthermore, because the combined therapy of Kv1.3 and KCa1.1 blockers was more beneficial than the monotherapies, it can be inferred that the monotherapies do not have large downstream effects on the reciprocal cell type through limiting either the T_EM_ cell-induced increase in FLS pathogenicity when blocking Kv1.3 or limiting the FLS-induced increase in T_EM_ cell pathogenicity when blocking KCa1.1. This also implies that both FLS and T_EM_ cells drive disease progression, as opposed to one cell type inducing the pathogenic phenotype of the other.

However, through examining the ex vivo invasiveness of FLS isolated from rats of each treatment group, we found that FLS from arthritic rats treated with ShK-186 had reduced invasion compared with FLS from vehicle-treated animals. FLS do not express Kv1.3, and ShK-186 does not block KCa1.1 and does not have an effect on FLS phenotypes [19, 31, 32]. This indicates that our observations in the present study were not a result of ShK-186 directly affecting FLS. Therefore, our data show that even though the FLS were not directly inhibited by ShK-186, the decrease in T_EM_ cell pathogenicity did have at least some downstream effects on FLS in vivo. Similarly, T_EM_ cells do not express KCa1.1, and paxilline and IbTX do not block Kv1.3 [7, 33, 34]. Therefore, the effects of KCa1.1 blockers in ameliorating disease in PIA and CIA were not due to directly inhibiting T_EM_ cells.

The populations of T cells within the draining inguinal lymph nodes of rats with CIA treated with potassium channel blockers were altered compared with those of vehicle-treated rats. For example, those treated with ShK-186, IbTX, or both ShK-16 and IbTX had an increase in the proportion of CD8^+^ T cells compared with those from vehicle-treated rats with CIA. CD8^+^ T cells may have a protective role in RA [68], and our data suggest that CD8^+^ T cells are correlated with a decreased disease burden. We also found large differences in the proportion of CD4^−^CD8^−^ T cells in the draining inguinal lymph nodes of rats with CIA, in which those treated with IbTX in the presence or absence of ShK-186 had dramatically reduced populations of these cells compared with vehicle-treated animals. Interestingly, although the proportion of these cells were decreased in these rats, they expressed more of the activation marker CD25 than vehicle-treated rats with CIA. The role of CD4^−^CD8^−^ T cells in RA and the roles of Kv1.3 and KCa1.1 in them is not well understood. Overall, our studies suggest that both Kv1.3 and KCa1.1 blockers affect T cell populations in CIA, though the mechanisms by which they do so, along with the roles of some of these cells, are yet to be investigated.

The ex vivo invasion of FLS is directly correlated with disease severity in patients with RA [37, 69, 70], and we found that FLS isolated from rats with PIA or CIA that were treated with Kv1.3 or KCa1.1 blockers, alone or in combination, exhibited reduced ex vivo invasion compared with vehicle-treated rats. These results agree with previous findings regarding FLS invasion as a measure of disease severity and joint destruction [69]. However, although the rats with PIA or CIA that were treated with a combined therapy of KCa1.1 and Kv1.3 blockers had reduced disease severity compared with monotherapy-treated rats, the ex vivo invasiveness was approximately the same. This may be due to limitations of the technique used to measure invasion, because the method we used involved measuring the number of cells that invaded through Matrigel at a single time point and did not account for differences in their rates of invasion.

## Conclusions

Overall, these studies provide further insights into the role of FLS and T cell interactions during RA and the importance of the potassium channels these cells express as mediators of these interactions. We also validated a novel therapeutic approach to treating RA by simultaneously inhibiting FLS and T_EM_ cells through targeting the predominant potassium channels by which these cells are regulated. In doing so, we further validated the central role of FLS and T_EM_ cells in the pathogenesis of RA and the importance of KCa1.1 and Kv1.3 in driving disease progression.

## Additional files

Additional file 1: **Table S1.** Serum chemical analyses of healthy rats and rats with CIA treated with vehicle, IbTX, ShK-186, or IbTX and ShK-186 every other day. Data are shown as mean (SD). *n* = 5 rats per group. (DOCX 17 kb)

Additional file 2: **Table S2.** Serum cytokine analyses of healthy rats and rats with CIA treated with vehicle, IbTX, ShK-186, or IbTX and ShK-186 every other day. Data are shown as mean (SD). *n* = 5 or 6 rats per group. (DOCX 16 kb)

## Abbreviations

APC: Allophycocyanin
CFSE: Carboxyfluorescein succinimidyl ester
CIA: Collagen-induced arthritis
FLS: Fibroblast-like synoviocytes
IbTX: Iberiotoxin
ICAM-1: Intercellular adhesion molecule 1
IFN: Interferon
KCa1.1: Large-conductance calcium-activated potassium channel 1.1
Kv1.3: Voltage-gated potassium channel 1.3
MHC: Major histocompatibility complex
Pax: Paxilline
PIA: Pristane-induced arthritis
RA: Rheumatoid arthritis
RANKL: Receptor activator of nuclear factor κΒ ligand
T_EM_ cell: Effector memory T cell
TNF: Tumor necrosis factor
