# Supplementary material for: KCa1.1 and Kv1.3 channels regulate the interactions between fibroblast-like synoviocytes and T lymphocytes during rheumatoid arthritis
Source: Arthritis Res Ther. 2019 Jan 7;21:6. doi: 10.1186/s13075-018-1783-9 (PMC6322314; doi:10.1186/s13075-018-1783-9)
Supplement: Supplementary file 1 — Table S1. Serum chemical analyses of healthy rats and rats with CIA treated with vehicle, IbTX, ShK-186, or IbTX and ShK-186 every other day. Data are shown as mean (SD). n = 5 rats per group. (DOCX 17 kb) [file 13075_2018_1783_MOESM1_ESM.docx]

|  | Healthy | CIA,  Vehicle | CIA,  ShK-186 | CIA,  IbTX | CIA,  ShK-186 + IbTX |
| --- | --- | --- | --- | --- | --- |
| Sodium  (mmol/L) | 127.4  (0.9) | 131.6  (0.5) | 132.4  (1.1) | 131.6  (1.1) | 136.2  (3.6) |
| Potassium  (mmol/L) | 3.72  (0.08) | 4.56  (0.15) | 4.2  (0.1) | 4.68  (0.74) | 4.92  (0.47) |
| Chloride  (mmol/L) | 96.2  (1.1) | 100  (1) | 98.2  (0.8) | 98.2  (1.3) | 105.8  (3.9) |
| CO2  (mmol/L) | 24.2  (0.4) | 20  (1.2) | 20.6  (1.1) | 18.8  (2.6) | 23  (1.2) |
| Albumin  (g/dL) | 4.7  (0.4) | 3.02  (0.2) | 3.54  (0.34) | 3.76  (0.13) | 3.8  (0.35) |
| CREA  (mg/dL) | 0.1  (0) | 0.1  (0) | 0.14  (0.05) | 0.14  (0.05) | 0.16  (0.05) |
| Total Protein  (g/dL) | 6.12  (0.56) | 4.7  (0.3) | 5.4  (0.27) | 5.66  (0.21) | 5.78  (0.37) |
| Globulin  (g/dL) | 1.44  (0.21) | 1.68  (0.18) | 1.84  (0.34) | 1.92  (0.22) | 1.98  (0.04) |
| A/G Ratio | 3.24  (0.18) | 1.82  (0.31) | 1.94  (0.42) | 2  (0.28) | 1.9  (0.17) |
| ALT  (U/L) | 28  (3.39) | 38  (5.70) | 35.2  (8.41) | 50  (13.91) | 50.6  (27.91) |
| AST  (U/L) | 58.4  (14.3) | 99.8  (19.3) | 88.6  (25.8) | 87.2  (21.8) | 95  (28.5) |
| ALP  (U/L) | 85.8  (13.3) | 131.6  (17.3) | 136.8  (18.3) | 143  (18.7) | 150.4  (34.8) |
| CK  (U/L) | 203.6  (89.9) | 381.6  (165.7) | 306.2  (202.3) | 370.4  (335.1) | 287.6  (153.7) |
| GGT  (U/L) | 0  (0) | 0  (0) | 0  (0) | 0  (0) | 0  (0) |
| LDH  (U/L) | 71.2  (48.3) | 478.8  (113.6) | 389.4  (203.9) | 470.8  (257.9) | 496.8  (134.2) |
| BUN  (mg/dL) | 9.46  (0.76) | 11.02  (0.73) | 10.2  (2.19) | 10.68  (1.08) | 11.12  (1.28) |
| Glucose  (mg/dL) | 102.2  (22.1) | 115.6  (13.8) | 112  (17.9) | 111  (14.9) | 119.2  (4.5) |
| Osmolality | 255.18  (1.55) | 264.16  (1.63) | 265.14  (2.65) | 263.76  (2.91) | 273  (6.74) |
| Bilirubin direct  (mg/dL) | 0.01  (0.01) | 0.002  (0.004) | 0.008  (0.008) | 0.01  (0.01) | 0.002  (0.004) |
| Indirect Bilirubin | 0  (0) | 0  (0) | 0  (0) | 0  (0) | 0.04  (0.05) |
| Cholesterol  (mg/dL) | 90.4  (8.0) | 74  (4) | 77.4  (5.6) | 84  (9.2) | 84  (5.1) |
| Triglycerides  (mg/dL) | 222.4  (49.9) | 43.8  (18.2) | 41.2  (15.6) | 65.8  (39.1) | 61.4  (24.6) |
| VLDL  (mg/dL) | 44.5  (9.95) | 8.76  (3.65) | 8.2  (3.13) | 13.2  (7.82) | 12.4  (4.87) |
| Magnesium  (mg/dL) | 1.7  (0.2) | 1.74  (0.05) | 1.84  (0.17) | 1.9  (0.07) | 1.96  (0.09) |
| Phosphorus  (mg/dL) | 4.08  (0.26) | 5.54  (0.35) | 5.7  (0.51) | 6.02  (0.38) | 6.68  (0.70) |
| Calcium  (mg/dL) | 9.584  (0.546) | 8.678  (0.303) | 9.214  (0.275) | 9.924  (0.361) | 10.734  (0.212) |

**Table S1.** Serum chemistry analyses of healthy rats and of rats with CIA treated with vehicle, IbTX, ShK-186, or IbTX and ShK-186 every other day. Data are shown as mean (standard deviation). N = 5 rats per group.
