# Supplementary material for: KCa1.1 and Kv1.3 channels regulate the interactions between fibroblast-like synoviocytes and T lymphocytes during rheumatoid arthritis
Source: Arthritis Res Ther. 2019 Jan 7;21:6. doi: 10.1186/s13075-018-1783-9 (PMC6322314; doi:10.1186/s13075-018-1783-9)
Supplement: Supplementary file 2 — Table S2. Serum cytokine analyses of healthy rats and rats with CIA treated with vehicle, IbTX, ShK-186, or IbTX and ShK-186 every other day. Data are shown as mean (SD). n = 5 or 6 rats per group. (DOCX 16 kb) [file 13075_2018_1783_MOESM2_ESM.docx]

|  | Healthy | CIA,  Vehicle | CIA,  ShK-186 | CIA,  IbTX | CIA,  ShK-186 + IbTX |
| --- | --- | --- | --- | --- | --- |
| Eotaxin | 4.51  (0.84) | 6.13  (3.77) | 5.53  (1.26) | 6.06  (1.64) | 4.38  (2.14) |
| EGF | 0  (0) | 0  (0) | 0  (0) | 0.01  (0.01) | 3.61  (8.84) |
| Fractalkine | 66.46  (8.20) | 36.49  (7.04) | 32.44  (8.13) | 39.27  (14.75) | 33.12  (12.05) |
| G-CSF | 31.74  (9.54) | 36.88  (23.00) | 34.50  (9.86) | 38.95  (16.37) | 25.06  (12.44) |
| GM-CSF | 14.91  (13.20) | 2.44  (5.46) | 0.54  (1.33) | 0.17  (0.40) | 0  (0) |
| GRO/KC | 154.71  (126.51) | 0  (0) | 0  (0) | 0  (0) | 0  (0) |
| IL-1α | 7.78  (3.86) | 32.96  (48.68) | 14.22  (5.43) | 13.33  (9.35) | 34.10  (52.78) |
| IL-1β | 58.05  (18.29) | 94.44  (76.73) | 49.75  (32.19) | 84.16  (90.11) | 54.28  (48.60) |
| IL-2 | 2.73  (1.49) | 91.71  (70.04) | 83.16  (57.63) | 100.17  (46.05) | 52.30  (66.82) |
| IL-4 | 6.84  (2.00) | 17.76  (7.50) | 13.82  (7.10) | 12.88  (2.36) | 12.81  (5.56) |
| IL-5 | 20.43  (5.45) | 65.43  (17.10) | 54.32  (16.16) | 54.02  (13.55) | 48.15  (24.90) |
| IL-6 | 455.87  (183.79) | 402.02  (236.03) | 233.01  (161.39) | 240.36  (259.74) | 395.48  (223.60) |
| IL-10 | 89.84  (32.89) | 101.90  (32.71) | 57.73  (19.50) | 108.57  (92.15) | 71.13  (52.95) |
| IL-12 | 10.12  (12.77) | 331.67  (192.45) | 220.20  (160.11) | 198.49  (84.32) | 225.57  (196.03) |
| IL-13 | 3.81  (1.37) | 9.19  (7.43) | 4.41  (2.50) | 4.88  (2.13) | 7.62  (7.51) |
| IL-17A | 7.56  (1.91) | 28.21  (11.49) | 20.68  (4.88) | 19.81  (5.64) | 14.74  (10.75) |
| IL-18 | 66.30  (13.17) | 156.11  (70.85) | 111.62  (48.31) | 142.30  (44.74) | 87.86  (58.76) |
| IFN-γ | 7.10  (5.99) | 3.45  (5.67) | 0  (0) | 0  (0) | 0.79  (1.92) |
| IP-10 | 267.01  (66.12) | 233.74  (47.81) | 211.07  (38.11) | 236.84  (64.00) | 230.14  (30.21) |
| Leptin | 10916.50  (5191.39) | 3109.75  (1432.88) | 2604.96  (1135.40) | 2967.42  (1538.39) | 4055.62  (2031.21) |
| LIX | 2486.88  (155.23) | 1476.93  (351.59) | 1653.69  (172.88) | 1590.57  (716.62) | 1237.78  (246.29) |
| MCP-1 | 968.57  (145.26) | 1339.39  (319.37) | 1078.18  (117.20) | 1017.76  (61.88) | 992.24  (256.24) |
| MIP-1α | 12.01  (1.12) | 11.40  (2.51) | 8.33  (1.59) | 9.71  (1.39) | 7.96  (1.79) |
| MIP-2 | 44.46  (17.12) | 0  (0) | 0  (0) | 10.35  (25.36) | 0  (0) |
| RANTES | 3149.22  (802.19) | 1131.65  (367.89) | 1821.84  (737.55) | 1638.91  (773.41) | 1517.23  (515.65) |
| TNF-α | 3.57  (2.18) | 18.43  (14.70) | 11.86  (6.14) | 13.17  (4.65) | 8.03  (7.13) |
| VEGF | 48.77  (5.23) | 39.57  (6.79) | 32.25  (6.32) | 43.82  (15.57) | 26.62  (15.94) |

**Table S2.** Serum cytokine analyses of healthy rats and of rats with CIA treated with vehicle, IbTX, ShK-186, or IbTX and ShK-186 every other day. Data are shown as mean (standard deviation). N = 5-6 rats per group.
